# Intelligent Topical Sentiment Analysis for the Classification of E-Learners and Their Topics of Interest

**DOI:** 10.1155/2015/617358

**Published:** 2015-03-18

**Authors:** M. Ravichandran, G. Kulanthaivel, T. Chellatamilan

**Affiliations:** ^1^Department of Computer Science and Engineering, Sathyabama University, Tamil Nadu 600119, India; ^2^Educational Media Centre, NITTTR, Chennai 600113, India; ^3^Department of Computer Science and Engineering, Arunai Engineering College, Tiruvannamalai 606603, India

## Abstract

Every day, huge numbers of instant tweets (messages) are published on Twitter as it is one of the massive social media for e-learners interactions. The options regarding various interesting topics to be studied are discussed among the learners and teachers through the capture of ideal sources in Twitter. The common sentiment behavior towards these topics is received through the massive number of instant messages about them. In this paper, rather than using the opinion polarity of each message relevant to the topic, authors focus on sentence level opinion classification upon using the unsupervised algorithm named bigram item response theory (BIRT). It differs from the traditional classification and document level classification algorithm. The investigation illustrated in this paper is of threefold which are listed as follows: (1) lexicon based sentiment polarity of tweet messages; (2) the bigram cooccurrence relationship using naïve Bayesian; (3) the bigram item response theory (BIRT) on various topics. It has been proposed that a model using item response theory is constructed for topical classification inference. The performance has been improved remarkably using this bigram item response theory when compared with other supervised algorithms. The experiment has been conducted on a real life dataset containing different set of tweets and topics.

## 1. Introduction

Social network analysis (SNA) can be considered as a global methodological approach to measure, visualize, and predict the interaction with one another in their field of study.

The learning relationship between the students from their similar cultural background and their topic of interest can be analyzed. The social network environment helps e-learning system through different ways.

1. SNA and text mining techniques can be applied to perform topical modeling and knowledge extraction.
2. The various online data mining techniques like classification, clustering information retrieval, question answering system, and query expansion are being used for social network analysis to the e-learning environment.
3. The learning community can recommend the learning material through collaborative recommendation globally based on their common interests related to their learning style, topic of study, and learning goal.
4. The international students learn and interact with each other through various aspects of a host culture. Detection of teaching community is related to the respective field of study.
5. The sentiment analysis can be applied to various interactions between the learners to build and then predict the model for reflecting the common interestingness through passing the comments (tweets) and feedbacks (retweets) of the learners.
6. The teaching learning process can be analyzed by the cognitive sequence and opinion of various learners.

The labeling accuracy of unlabeled samples is improved by generating distribution feature of emotional strength. The distribution view of dimensionality is used for adaptive emotion recognition of posts in the online student community [1]. The semantic meaning of documents and images is analyzed using latent topical models with only partial labels in the training process. The topic model method ensures that the learned topic has good relation with class labels [2]. It has been observed that the emotional intelligence (EI) of the teacher has a significant impact on teaching satisfaction [3]. The emotional intelligence helps the teacher and student to predict their performance. The emotional skills of teachers influence how students behave, engagement and attachment to school, and their academic performance [4]. The emotional intelligence is also useful for the student-teachers needs [5]. There is a positive significant correlation between perceived EI and self-efficacy [6]. The parental involvement in monitoring homework and participation in extracurricular activities and the parent teachers association are also significant in e-learning environment [7]. The student's ability/performance and achievement are strongly related to human cognitive social emotions. Research experiments are conducted to find the correlation among these things [8]. The topic identification is a method which interprets large number of tweet messages and then estimates the score of the interestingness of each of the tweets based on the latent topic [9]. In the supervised learning, weighting scheme gives the importance to a term in a document [10]. SentBuk is a Facebook application which receives messages written by users and classifies them according to its polarity through the interactive interface. In the field of e-learning, it is very useful to have information about the user's sentiments to support personalized learning [11]. The emotional understanding of the various users can be analyzed through Facebook sharing of small stories and narrative statements [12]. The EI helps the information search in tactical decision making [13]. IRT is used in the development of performance based digital test and the validation of a direct measure of ICT competence [14]. The corpus based and dictionary based methods have been proposed to determine the semantic orientation of opinion words in tweets [15]. The tweet sampling algorithms automatically monitor the target tweet from the twitter stream for any given topic based on keyword extraction technique [16]. The probabilistic topic modeling helps in the recommendation of newly published articles to the readers [17].

### 1.1. Novelty of Topical Sentiment Analysis

The novel algorithm called bigram topical item response theory (BIRT) for sentiment classification is achieved by an objective function which builds the model for the representation and then predicts the document sentiment. The design of adaptive text paper and education assessment based on IRT (item response theory) is proposed [18, 19].

The topical sentiment analysis recognizes the polarity of opinion and emotion attributes regarding the topic of interest. The subjectivity strongly depends on its sentences or messages. The novelty of lexicon and its semantics can be used to distinguish between words and phrases in determining the polarity of the sentiment [20, 21]. The modified item response theory helps to achieve personalized learning and provide learning pathways and helps them to learn effectively [22, 23]. The novelty of the proposed method is that the students' tweets are not simply classified by sentiment polarity but instead generate the grading of sentiment for each selected topic. In this paper rather than using the opinion polarities of each message relevant to the topic, the sentence level opinion classification based on BIRT is discussed. Unlike the fixed set of responses, dynamic response theory in terms of multiple factors on varied topics by different sets of interactions between the user communities offers the novelty of sentiment analysis.

In the previous study, the problem of classification deals with traditional statistical and probability distribution for scaling the polarity. The mapping from the real line is provided through the probability interval 0 to 1. Using such logit functional model the real time traffic crash can be predicted using the traffic-flow variables and rain data [24]. The bias component can be incorporated into the classification with the help of the IRT model which in turn generalizes the data and minimizes cost of parameter estimation. The likelihood of the responses and item level analysis can be formulated through the IRT model.

## 2. Sentiment Analysis

There are two important steps for social media mining:

1. retrieval of content related to the topic of interest;
2. measurement of the polarity of each tweet of the topic of interest.

In the first step the sentiment content related to particular topic should be sleeked through the respective tweets. Topic models represent more sophisticated and potentially move through way of capturing bits of text that are relevant to a practical analysis. These models are constructed by lacing very large dataset of documents as the inputs and clustering them into estimated topics of probability.

In the machine learning approach the sentiment analysis is a kind of text classification [25] and it can be solved by training the classifier on a labeled text collection. The hierarchical generative probabilistic model incorporates both N-gram principles and latent topic variables by modifying the unigram topic model [26]. The graph based hashtag sentiment classification is applied to estimate the score of the polarity [27]. An investigation study has been conducted for the postreading activities of community college students in twitter environment for language learning [28]. The interestingness of the individual learner has been found out using their tweet, retweet, and content link details with the help of topic modeling and topical analysis [29]. In order to identify topics in the tweets and articles during the recommendation, the semantic enrichment component is required for user modeling [30]. Vent discovery is another field of social media mining to examine and share an unfolding life event across the Facebook users where the posts are arranged in chronological order [31]. A hybrid text based and community based user profiling system estimates the behavior of the user by tracking the tweet history and their followers and followee [32]. Emotional intelligence shows positively/negatively influencing mood of the patients in predicting their health behavior, accounted by similar patients [33]. Item response theory models perform the relative parameter estimation from the response data available in the corpus [34]. The important subjective portion of the documents was extracted by finding the minimum cross cut graph mining technique using the respective sentiment polarity [35]. Student knowledge tracing process allows the prediction of efficient features in the classification of students and their performance [36]. A case based reasoning guides the learner to regulate the negative emotion of the teacher through sentence similarity computation [37]. The R statistical programming language provides the framework for modeling latent variable multivariate analysis [38].

## 3. General Architecture

**Figure 1:**
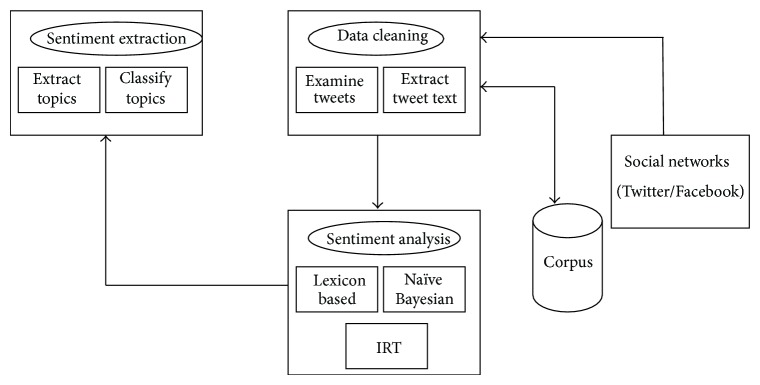
General architecture for topical classifications.

Figure 1 shows the general framework of our proposed approach. The different components involved in this framework are explained in the subsections.

### 3.1. Data Cleaning

After tweets are gathered from the social network using twitter API based on the query string hash tags, we prepared dataset for sentiment analysis.

1. Collect the tweets that are describing a particular topic from the dataset.
2. Remove retweet entities, URL removal, markup removal, and hash tags removal.

For each given set of tweets, we removed punctuation, numbers, white spaces, and unnecessary symbols.

### 3.2. Topic Extraction and Matching

To reveal the sentiment of each tweet based on the topic associated with sentiment of each user, the topical words (bigram) are extracted from the tweet messages using item response theory and are categorized according to its unsupervised nature of the features. Topic proportion exhibits the sentiment, according to the logistic and the latent trait structure model of each tweet user with the ability level of the user particular to a topic. Idea behind the algorithm is to find those terms that relate to a topic sentiment with respect to the topic sentiment lexicon.

### 3.3. Lexicon Based Approach

This approach measures the sentiment by few curving lexicons agreement. Our tweets message to be tested on topic models. Because the entire document is the mixture of one or more topics which are estimated using the parameter estimation technique, this allows the users to find the text that is relevant to the topic through the use of a particular keyword. The lexicon approach measures the sentiment of a group of document corpus with the help of dictionary of words and its associated polarity scores in the training corpus and all such words of the documents are compared to the word usage in the lexicon. There are many ways generally for the lexicon to offer best chance to successfully estimate sentiment:

- preassembly domain specific lexicon;
- dictionary based lexicon;
- corpus based lexicon.

The dictionary of lexicons elements is added externally to the corpus for the purpose of enhancing the preassembled lexicons. In the lexicon based sentiment analysis, it is sufficient to simply count the term frequency of every document relevant to our topic of interest. The conditional probabilities of each and every lexical token in the vocabulary were computed with the help of training sample using the following equation:

(1) $$\begin{array}{c} P\left(w\;|\;+\right)=\frac{M_{w}}{\left|N^{+}\right|},\\ P\left(w\;|\;-\right)=\frac{M_{w}}{\left|N^{-}\right|}. \end{array}$$

The score of positive and negative sentiment is coded as

(2) $$\begin{array}{c} N^{+},N^{-}. \end{array}$$

For each sentence message “*m*,” the log likelihood ratio is calculated using the following equation:

(3) $$\begin{array}{c} S_{m}=\overset{n}{\underset{i=1}{\sum }}\log\left(\frac{P\left(w_{i}\;|\;-\right)}{P\left(w_{i}\;|\;+\right)}\right), \end{array}$$

where  *w* is the lexical unit of the dictionary and *n* is the number of words and collocations included in the dictionary, which are found in the sentence “*m*.” *M*_*w*_ is the set of messages containing lexical token “*w*.”

**Algorithm 1 .**

1. Download the +ve and −ve lexicons from the web portal.
2. Generate the lexicons based on the preassemble (download) dictionary based and corpus based approach.
3. Preprocess and construct extracted tweet messages.
4. Create a corpus using the tweet vector source.
5. Clean the data by stop word removal stemming punctuation removal.
6. Consult term document matrix and sort the frequent words that leak dictionary power as a consequence of their repeated use.
7. Find out the most frequent words against the polarity of the sentiment analysis.
8. Calculate the mean score of each polarity.
9. Display classification between using histogram of raw source and centered score to verify the impact of polarity classifications.

### 3.4. Naïve Bayes Classifier: Lexical/Token Level Classifier

It is a supervised approach of classification that utilizes the data that has been tagged or labeled. It involves a training dataset of documents that have been already scored as positive or negative sentiment. The labeled dataset is mandatory for applying this kind of classifier. To do so a large set of documents are already coded as containing positive and negative sentiments about predicting the direction of the sentiment or valence.

The probability of a word or a term for the given class (polarity) is obtained by

(4) $$\begin{array}{c} P\left(t\;|\;c\right)=\frac{T_{\text{ct}}+1}{\sum_{t\in V}\left(T_{\text{ct}}+1\right)},\\ c_{\text{map}}=\underset{c\in C}{\text{argmax}}\left(\log P\left(c\right)+\overset{n}{\underset{k=1}{\sum }}\log P\left(t_{k}\;|\;c\right)\right),\\ c_{\text{map}}=\underset{c\in C}{\text{argmax}}\left(P\left(c\;|\;d\right)\right),\\ c_{\text{map}}=\underset{c\in C}{\text{argmax}}\left(P\left(c\right)\underset{1\leq k\leq n}{\prod }P\left(t_{k}\;|\;c\right)\right), \end{array}$$

where *d* is the document in the corpus, *T*_ct_ is the term count in the document of class *c*, *P* is the probability, *V* is the vocabulary, *t* is the term, *c* is the class, and *n* is the total number of terms in the vocabulary.

**Algorithm 2 .**

1. Generate the dataset from tweets.
2. Drop unnecessary variables from the tweets.
3. Create a corpus by removing punctuation stop words whitespace and stemming.
4. Create a document term matrix for the trigram of tweets (using weak).
5. Drop uses tweet that are very small number of trigrams (least common trigram).
6. Construct model as an object called NB model and run the model.
7. Upon using this model the text data has been applied for the prediction of its sentiments polarity.

### 3.5. Item Response Theory Classifier: Bigram/Sentence Level Classifier

It is a family of statistical theory which measures more preciously psychometric measurements. The psychometric assignment maps the observations onto internal states or traits. The output of the measurement is mapped to the unobserved trait using some scaling rules. IRT makes explicit the assumptions required to justify and to make inference about the latent qualitative parameters.

The supervised classifiers are working good under a wide verity of conditions such as heterogeneous text length and topic breath with large amount of labeled training data for the substantial classification. IRT is an unsupervised approach of statistical model. One of the strong assumptions in IRT is that the text-topic is a sequence of long continuum of words whereas the importance of a word token highly depends on its cooccurrence word and its position. The tweet set is such that dataset represents the underlying continuum of sentiment about a single moderately narrow topic and that continuum likely affects the word choice of each document. Item response theory has distinct feature and adopts probabilistic model of each possible response to a test case. IRT derives the probability of each response as a procedure of the hidden trait and some item features. The IRT model helps to find likelihood value of observed responses (estimation). Using IRT model, the probability of topical word belongs to a class that is correctly classified with its class using the following formula:

(5) $$\begin{array}{c} P_{j}\left(\alpha \right)=\frac{e^{\left(\alpha -a_{j}\right)}}{\left(1+e^{\left(\alpha -a_{j}\right)}\right)}, \end{array}$$

where *p*_*j*_(*α*) is the probability of randomly selected words or tokens with ability *α* being its correct class of the *j*th word. *e* is the natural log. *α* is the ability level of classification in logit. *a*_*j*_ is the item parameter. Difficulty features the word item measured in logit.

IRT basically equates the ability of the classifier with the difficulty of the test attributes during classification. IRT model can have the power to perform computation with multiple number of parameters with probability theory. The IRT models having one, two, or three parameters and their probability predicted by the model were represented as *p*_*ij*_(*α*_*j*_, *a*_*i*_), *p*_*ij*_(*α*_*j*_, *a*_*i*_, *b*_*i*_), and *p*_*ij*_(*α*_*j*_, *a*_*i*_, *b*_*i*_, *c*_*i*_), where *a*_*i*_, *b*_*i*_, and *c*_*i*_ are item features.

**Algorithm 3 .**

1. Collect tweets and construct the dataset.
2. Construct document term matrix of the tweets corpus.
3. Aggregate all the tweets by its user(learner/teacher) and then convert it into document term matrix.
4. Consider the matrix as one row per user instead of one row per tweet. IRT assumes that all of word choices are affected by his position on the underlying condition.
5. Aggregate our label vector which we will use for model checking later.
6. DNP users who use a very small number of bigrams, keeping only the users who employ at least 4 key bigrams, make a good chord.
7. Estimate the logistic model.
8. Capture the scale position of user for whom we had enough data to scale.
9. Draw the histogram of overall distribution of position in the data.
10. Generate the list of big words which are most discriminating bigram.
11. Accuracy of the model is shown in the proofs.

This paper presents an alternative method of estimating latent variables using models from IRT. The key strength of the IRT approach is that the latent variables are an explicit part of the model and parameters to be estimated, rather than mere byproducts of a data reduction exercise (as in conventional factor analytic approaches). Item-response modeling, on the other hand, directly models the individual responses on the observed variables, with individual scores on the latent factors as parameters to be estimated, as well as the equivalent of factor loadings. In this way, IRT is widely considered a more principled approach to measurement, which modern computing power now allows us to exploit.

## 4. Experiments and Results

The Stanford Gold Dataset of 498 tweets was scraped. In the first step we have applied different data cleaning processes like dropping punctuation, changing to lower case, and stemming. Next the bigram doc-term matrix was created for each of the tweets. Finally the tweets are aggregated up to level of users and then we removed bigram used by only one user and removed users with 1 or less bigram. Using this result set, a model is constructed using ideal function in the pscl package in R. We plot the results in three graphs as shown.

**Figure 2:**
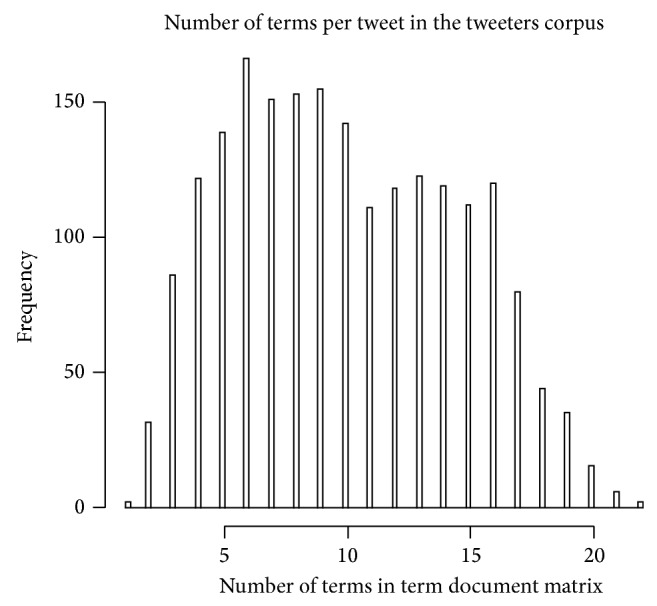
Term frequency of the corpus.

The histogram in Figure 2 consists of parallel vertical bars that graphically show the frequency distribution of a quantitative term frequency of the word. The area of each bar is equal to the frequency of items found in the corpus.

**Figure 3:**
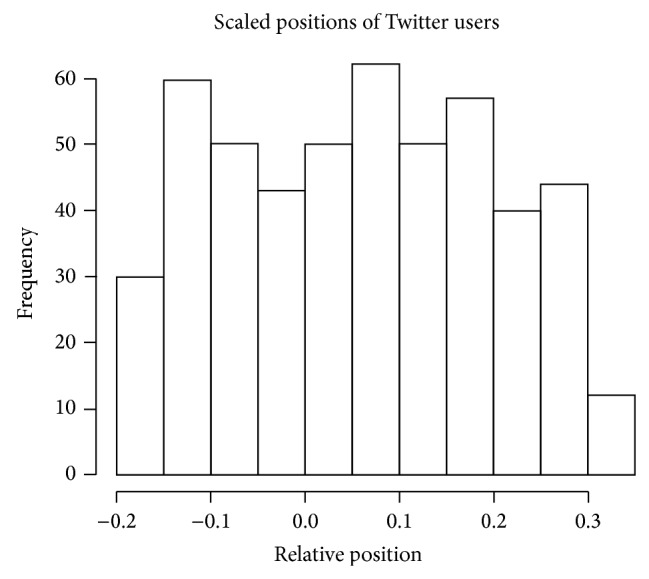
Scaled position of learners.

The histogram in Figure 3 shows the overall distribution of positions in the data. Foremost, these values are relative to one another. The numbers on *x*-axis describe the distance between the learners, but not compared to any true values. This means that zero, rather than meaning neutral, probably means something closer to central. Similarly, the score only captures the fact that a learner at zero is to the left of learners at 0.1 and to the right of learners at −0.1. We still would have had a distribution centered at zero, even though all of the tweets being scaled would be to the right of center.

**Figure 4:**
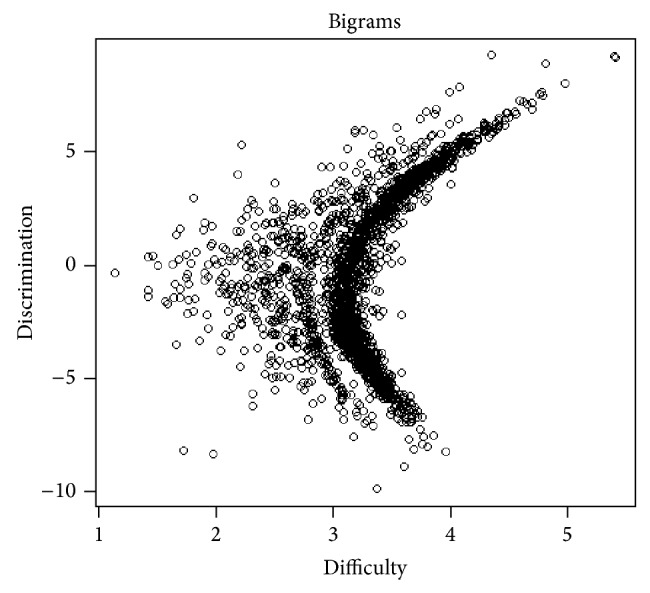
Term frequency of the corpus.

People at the same end of the scale should have the same views, generally. If not, the model may have failed or pulled out an underlying continuum other than the one we were looking for. Also, we may be interested in the difficulty and discrimination parameters of each bigram. We can get a sense of both by plotting one against the other as shown in Figure 4.

**Figure 5:**
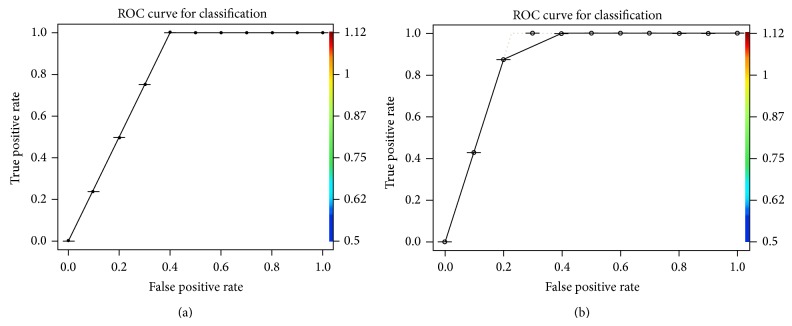
(a) ROC curve for naïve Bayes classifier. (b) ROC curve for BIRT.

Each point in the graph represents a bigram, whereas the *x*-axis represents the difficulty level of the bigram topic (measures of bigram rarity) and the *y*-axis plots each bigram's discrimination. It shows the evidence that how for it is more likely to be used by those learners on one side of the scale or the other learners of another side. For example, the large numbers of bigrams with positive discrimination parameter are likely to be used by those learners on the right side of the scale and unlikely to be used by those on left hand scale. The polarity signs + and − determine left and right, respectively, and the magnitude represents how strong the effect was. The equally used bigrams by all users are nearly zero discrimination level on all parts of the scale. While observing this graph, it has been observed that most bigrams are not discriminating between sides of the scale. The strong corelation exists between difficulty and discrimination. Most frequently used bigrams are not discriminating much whereas the infrequently used bigram discriminates better. We have put the results of this in the following graph named receiver operating characteristics (ROC), for showing the accuracy of the two classifiers named naïve Bayesian and IRT, respectively, through Figures 5(a) and 5(b); the *x*-axis here represents the false positive rate (FPR), while the *y*-axis represents the true positive rate (TPR). The IRT model seems to have scaled most of these tweets accurately. However, the model has a tough time discriminating authors near the middle of the scale, which is common in scaling applications.

### 4.1. Comparative Analysis

**Figure 6:**
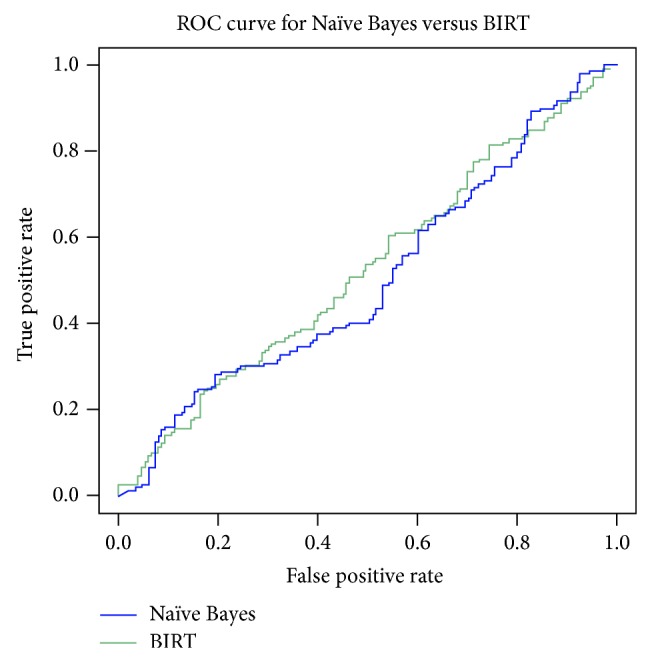
ROC for BIRT and naïve Bayes classifier.

Several conclusions can be drawn when we apply different classification algorithms over the text data. In the lexical token level classifier, the use of stop word removal and stemming degrades the performance of classifier. The bigram sentence level classifier uses bigram tokens in the bag of words, which gives additional information of multinomial in predicting the class label for classification. The BIRT outperforms the naïve classifier by applying the F1-measure and recalling shown in Figure 6.

## 5. Conclusion and Future Work

The learners/teacher's annotated topics are viewed as important for sentiment analysis. The simple voting strategy methodology named item response theory regarding the bigram tokenizing baseline is built. This approach efficiently incorporates the user's instant messages, topics, and then cooccurrence relationship. We construct the enhanced unsupervised classification framework IRT in which the self-expandable topic is labeled as hash and not involving the dynamic updating of polarity. Significant improvements are shown in the experimental result. The possible extensions can be made by producing the short summary of topics based on the sentiment classifications. The dynamic topic modeling can be used for improving the dynamic behavior of the content change in the tweet interaction of the users. The ontology based domain specific knowledge can be incorporated to improve the performance of the topical classification.
